# Supplementary material for: The Impact of Social Determinants of Health on Metabolic Dysfunction-Associated Steatotic Liver Disease Among Adults in the United States
Source: J Clin Med. 2025 Aug 4;14(15):5484. doi: 10.3390/jcm14155484 (PMC12347937; doi:10.3390/jcm14155484)
Supplement: Supplementary file 1 [file jcm-14-05484-s001.zip › jcm-3751012-supplementary.pdf]

Supplementary Figure and Table Legend

Figure S1. Prevalence of metabolic dysfunction-associated steatotic liver disease (MASLD) at different education levels

Figure S2. Prevalence of metabolic dysfunction-associated steatotic liver disease (MASLD) at different income brackets

Table S1A. Logistic regression model evaluating the association between education level and metabolic dysfunction-associated steatotic liver disease (MASLD), adjusted for sociodemographic factors (Model 1)

Table S1B. Logistic regression model evaluating the association between education level and metabolic dysfunction-associated steatotic liver disease (MASLD), adjusted for covariates in Model 1 plus medical comorbidities (Model 2)

Table S1C. Logistic regression model evaluating the association between education level and metabolic dysfunction-associated steatotic liver disease (MASLD), adjusted for covariates in Model 2 plus SDOH variables (Model 3)

Table S2A. Logistic regression model evaluating the association between income bracket and metabolic dysfunction-associated steatotic liver disease (MASLD), adjusted for sociodemographic factors (Model 1)

Table S2B. Logistic regression model evaluating the association between income bracket and metabolic dysfunction-associated steatotic liver disease (MASLD), adjusted for covariates in Model 1 plus medical comorbidities (Model 2)

Table S2C. Logistic regression model evaluating the association between income bracket and metabolic dysfunction-associated steatotic liver disease (MASLD), adjusted for covariates in Model 2 plus SDOH variables (Model 3)

Table S3A. Logistic regression model evaluating the association between education level and income bracket and metabolic dysfunction-associated steatotic liver disease (MASLD), adjusted for sociodemographic factors (Model 1)

Table S3B. Logistic regression model evaluating the association between income bracket and metabolic dysfunction-associated steatotic liver disease (MASLD), adjusted for covariates in Model 1 plus medical comorbidities (Model 2)

Figure S1. Prevalence of metabolic dysfunction-associated steatotic liver disease (MASLD) at different education levels

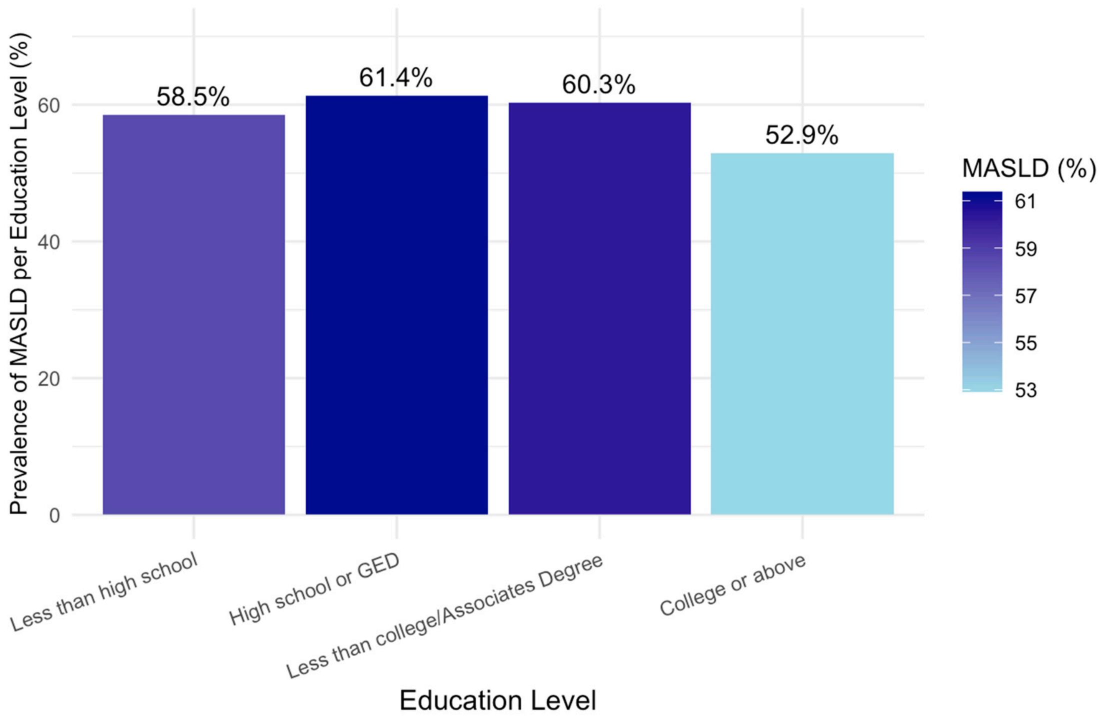

Figure S2. Prevalence of metabolic dysfunction-associated steatotic liver disease (MASLD) at different income brackets

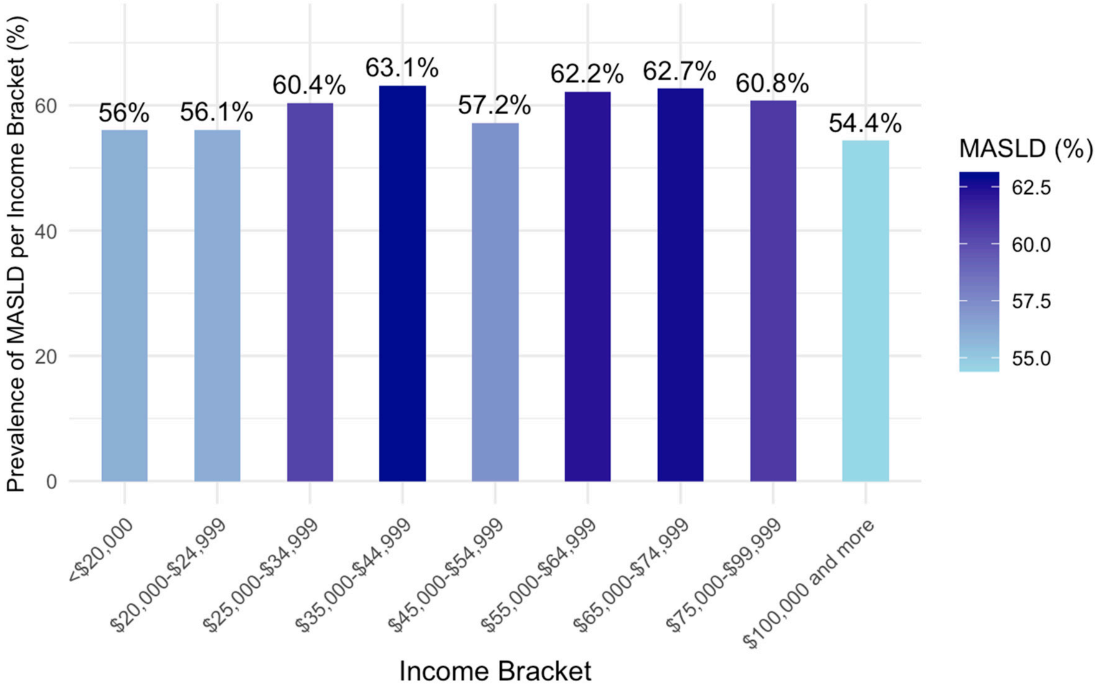

Supplementary Table 1A. Logistic regression model evaluating the association between education level and metabolic dysfunction-associated steatotic liver disease (MASLD), adjusted for sociodemographic factors (Model 1). *OR: Odds ratio; CI: Confidence interval.*

	**Univariable**			**Multivariable**		
	**OR**	**95% CI**	***p* value**	**OR**	**95% CI**	***p* value**
Education						
<College	*Ref*					
College or above	0.74	0.63, 0.86	<0.001	0.75	0.63, 0.90	0.002
Age	1.02	1.02, 1.03	<0.001	1.02	1.02, 1.03	<0.001
Male	1.4	1.22, 1.62	<0.001	1.35	1.17, 1.57	<0.001
Race/Ethnicity						
Non-Hispanic White	*Ref*					
Mexican American	1.74	1.38, 2.21	<0.001	1.95	1.53, 2.50	<0.001
Other Hispanic	1.12	0.85, 1.47	0.4	1.16	0.88, 1.54	0.3
Non-Hispanic Black	0.74	0.62, 0.89	0.001	0.77	0.64, 0.94	0.008
Non-Hispanic Asian	0.95	0.75, 1.21	0.7	1.16	0.90, 1.50	0.3
Other race	0.93	0.68, 1.27	0.6	1.06	0.77, 1.47	0.7

Supplementary Table 1B. Logistic regression model evaluating the association between education level and metabolic dysfunction-associated steatotic liver disease (MASLD), adjusted for covariates in Model 1 plus medical comorbidities (Model 2). *OR: Odds ratio; CI: Confidence interval; BMI: Body mass index.*

	Univariable			Multivariable		
	**OR**	**95% CI**	***p* value**	**OR**	**95% CI**	***p* value**
Education						
<College	*Ref*					
College or above	0.74	0.63, 0.86	<0.001	0.75	0.61, 0.92	0.005
Age	1.02	1.02, 1.03	<0.001	1.02	1.01, 1.03	<0.001
Male	1.4	1.22, 1.62	<0.001	1.41	1.19, 1.67	<0.001
Race/Ethnicity						
Non-Hispanic White	*Ref*					
Mexican American	1.74	1.38, 2.21	<0.001	1.7	1.29, 2.26	<0.001
Other Hispanic	1.12	0.85, 1.47	0.4	1.1	0.80, 1.51	0.6
Non-Hispanic Black	0.74	0.62, 0.89	0.001	0.59	0.47, 0.74	<0.001
Non-Hispanic Asian	0.95	0.75, 1.21	0.7	2.42	1.79, 3.28	<0.001
Other race	0.93	0.68, 1.27	0.6	0.99	0.68, 1.45	>0.9
Hypertension History	2.4	2.06, 2.80	<0.001	1.33	1.09, 1.63	0.005
Diabetes History	3.31	2.62, 4.22	<0.001	1.73	1.31, 2.30	<0.001
Hypercholesterolemia History	1.81	1.56, 2.11	<0.001	1.08	0.89, 1.31	0.4
BMI Category						
18.5-24.9	*Ref*					
<18.5	0.28	0.07, 0.80	0.037	0.43	0.10, 1.24	0.2
25-29.9	4.6	3.74, 5.68	<0.001	4.34	3.46, 5.45	<0.001
≥ 30	13.7	11.1, 17.1	<0.001	17	13.4, 21.8	<0.001
History of Hepatitis B	1.21	0.60, 2.55	0.6	0.93	0.41, 2.18	0.9
History of Hepatitis C	0.64	0.39, 1.06	0.082	0.56	0.31, 1.02	0.06
Alcohol Use						
Never drink	*Ref*					
Drink daily	0.87	0.64, 1.18	0.4	1.53	1.06, 2.22	0.024
Drink few times/week	0.85	0.69, 1.06	0.2	1.24	0.95, 1.61	0.1
Drink few times/month	0.78	0.63, 0.96	0.021	0.97	0.74, 1.26	0.8
Drink few times/year	0.91	0.75, 1.11	0.4	1.05	0.83, 1.35	0.7

Supplementary Table 1C. Logistic regression model evaluating the association between education level and metabolic dysfunction-associated steatotic liver disease (MASLD), adjusted for covariates in Model 2 plus social determinants of health (SDOH) variables (Model 3). *OR: Odds ratio; CI: Confidence interval; BMI: Body mass index.*

	**Univariable**			**Multivariable**		
	**OR**	**95% CI**	***p* value**	**OR**	**95% CI**	***p* value**
Education						
<College	*Ref*					
College or above	0.74	0.63, 0.86	<0.001	0.79	0.64, 0.99	0.036
Age	1.02	1.02, 1.03	<0.001	1.02	1.01, 1.02	<0.001
Male	1.4	1.22, 1.62	<0.001	1.42	1.19, 1.70	<0.001
Race/Ethnicity						
Non-Hispanic White	*Ref*					
Mexican American	1.74	1.38, 2.21	<0.001	1.71	1.29, 2.29	<0.001
Other Hispanic	1.12	0.85, 1.47	0.4	1.08	0.79, 1.49	0.6
Non-Hispanic Black	0.74	0.62, 0.89	0.001	0.63	0.50, 0.79	<0.001
Non-Hispanic Asian	0.95	0.75, 1.21	0.7	2.4	1.77, 3.26	<0.001
Other race	0.93	0.68, 1.27	0.6	1.01	0.69, 1.48	>0.9
Hypertension History	2.4	2.06, 2.80	<0.001	1.33	1.09, 1.63	0.005
Diabetes History	3.31	2.62, 4.22	<0.001	1.69	1.28, 2.25	<0.001
Hypercholesterolemia History	1.81	1.56, 2.11	<0.001	1.08	0.89, 1.32	0.4
BMI Category						
18.5-24.9	*Ref*					
<18.5	0.28	0.07, 0.80	0.037	0.4	0.10, 1.17	0.14
25-29.9	4.6	3.74, 5.68	<0.001	4.34	3.46, 5.46	<0.001
≥ 30	13.7	11.1, 17.1	<0.001	16.6	13.0, 21.3	<0.001
History of Hepatitis B	1.21	0.60, 2.55	0.6	0.92	0.41, 2.16	0.8
History of Hepatitis C	0.64	0.39, 1.06	0.1	0.56	0.31, 1.03	0.1
Alcohol Use						
Never drink	*Ref*					
Drink daily	0.87	0.64, 1.18	0.4	1.55	1.07, 2.25	0.022
Drink few times/week	0.85	0.69, 1.06	0.2	1.27	0.97, 1.66	0.1
Drink few times/month	0.78	0.63, 0.96	0.021	0.99	0.76, 1.29	>0.9
Drink few times/year	0.91	0.75, 1.11	0.4	1.05	0.82, 1.35	0.7
Moderate physical activity	0.5	0.43, 0.59	<0.001	0.73	0.59, 0.89	0.002
Full food security	0.86	0.74, 0.99	0.039	0.78	0.65, 0.95	0.012
Private insurance	1.11	0.96, 1.27	0.2	1.22	1.02, 1.47	0.032
Access to healthcare	1.44	1.20, 1.72	<0.001	1.02	0.81, 1.28	0.9
Married/Living with partner	1.42	1.23, 1.64	<0.001	1.29	1.08, 1.54	0.005

Supplementary Table 2A. Logistic regression model evaluating the association between income bracket and metabolic dysfunction-associated steatotic liver disease (MASLD), adjusted for sociodemographic factors (Model 1). *OR: Odds ratio; CI: Confidence interval.*

	**Univariable**			**Multivariable**		
	**OR**	**95% CI**	***p* value**	**OR**	**95% CI**	***p* value**
Income						
<65,000	*Ref*					
≥$65,000	0.97	0.84, 1.12	0.7	1.01	0.87, 1.18	0.9
Age	1.02	1.02, 1.03	<0.001	1.02	1.02, 1.03	<0.001
Male	1.4	1.22, 1.62	<0.001	1.36	1.18, 1.57	<0.001
Race/Ethnicity						
Non-Hispanic White	*Ref*					
Mexican American	1.74	1.38, 2.21	<0.001	2.03	1.60, 2.60	<0.001
Other Hispanic	1.12	0.85, 1.47	0.4	1.17	0.89, 1.54	0.3
Non-Hispanic Black	0.74	0.62, 0.89	0.001	0.78	0.65, 0.94	0.01
Non-Hispanic Asian	0.95	0.75, 1.21	0.7	1.04	0.81, 1.33	0.8
Other race	0.93	0.68, 1.27	0.6	1.08	0.78, 1.49	0.7

Supplementary Table 2B. Logistic regression model evaluating the association between income bracket and metabolic dysfunction-associated steatotic liver disease (MASLD), adjusted for covariates in Model 1 plus medical comorbidities (Model 2). *OR: Odds ratio; CI: Confidence interval; BMI: Body mass index.*

	**Univariable**			**Multivariable**		
	**OR**	**95% CI**	***p* value**	**OR**	**95% CI**	***p* value**
Income						
<65,000	*Ref*					
≥$65,000	0.97	0.84, 1.12	0.7	1.01	0.85, 1.21	0.9
Age	1.02	1.02, 1.03	<0.001	1.02	1.01, 1.03	<0.001
Male	1.4	1.22, 1.62	<0.001	1.41	1.19, 1.68	<0.001
Race/Ethnicity						
Non-Hispanic White	*Ref*					
Mexican American	1.74	1.38, 2.21	<0.001	1.78	1.35, 2.36	<0.001
Other Hispanic	1.12	0.85, 1.47	0.4	1.1	0.81, 1.52	0.5
Non-Hispanic Black	0.74	0.62, 0.89	0.001	0.59	0.48, 0.74	<0.001
Non-Hispanic Asian	0.95	0.75, 1.21	0.7	2.15	1.61, 2.89	<0.001
Other race	0.93	0.68, 1.27	0.6	1.01	0.69, 1.47	>0.9
Hypertension History	2.4	2.06, 2.80	<0.001	1.35	1.10, 1.65	0.003
Diabetes History	3.31	2.62, 4.22	<0.001	1.76	1.33, 2.33	<0.001
Hypercholesterolemia History	1.81	1.56, 2.11	<0.001	1.08	0.89, 1.31	0.5
BMI Category						
18.5-24.9	*Ref*					
<18.5	0.28	0.07, 0.80	0.037	0.44	0.10, 1.27	0.2
25-29.9	4.6	3.74, 5.68	<0.001	4.3	3.43, 5.41	<0.001
≥ 30	13.7	11.1, 17.1	<0.001	16.9	13.3, 21.6	<0.001
History of Hepatitis B	1.21	0.60, 2.55	0.6	0.95	0.42, 2.24	0.9
History of Hepatitis C	0.64	0.39, 1.06	0.1	0.58	0.32, 1.06	0.1
Alcohol Use						
Never drink	*Ref*					
Drink daily	0.87	0.64, 1.18	0.4	1.46	1.01, 2.13	0.044
Drink few times/week	0.85	0.69, 1.06	0.2	1.19	0.92, 1.55	0.2
Drink few times/month	0.78	0.63, 0.96	0.021	0.94	0.72, 1.22	0.6
Drink few times/year	0.91	0.75, 1.11	0.4	1.04	0.82, 1.33	0.7

Supplementary Table 2C. Logistic regression model evaluating the association between income bracket and metabolic dysfunction-associated steatotic liver disease (MASLD), adjusted for covariates in Model 2 plus social determinants of health (SDOH) variables (Model 3). *OR: Odds ratio; CI: Confidence interval; BMI: Body mass index.*

	**Univariable**			**Multivariable**		
	**OR**	**95% CI**	***p* value**	**OR**	**95% CI**	**p value**
Income						
<65,000	*Ref*					
≥$65,000	0.97	0.84, 1.12	0.7	1.06	0.87, 1.30	0.5
Age	1.02	1.02, 1.03	<0.001	1.02	1.01, 1.02	<0.001
Male	1.4	1.22, 1.62	<0.001	1.43	1.20, 1.70	<0.001
Race/Ethnicity						
Non-Hispanic White	*Ref*					
Mexican American	1.74	1.38, 2.21	<0.001	1.76	1.33, 2.35	<0.001
Other Hispanic	1.12	0.85, 1.47	0.4	1.08	0.78, 1.49	0.6
Non-Hispanic Black	0.74	0.62, 0.89	0.001	0.63	0.50, 0.79	<0.001
Non-Hispanic Asian	0.95	0.75, 1.21	0.7	2.19	1.63, 2.95	<0.001
Other race	0.93	0.68, 1.27	0.6	1.01	0.69, 1.48	>0.9
Hypertension History	2.4	2.06, 2.80	<0.001	1.34	1.09, 1.63	0.005
Diabetes History	3.31	2.62, 4.22	<0.001	1.7	1.29, 2.27	<0.001
Hypercholesterolemia History	1.81	1.56, 2.11	<0.001	1.09	0.89, 1.32	0.4
BMI Category						
18.5-24.9	*Ref*					
<18.5	0.28	0.07, 0.80	0.037	0.41	0.10, 1.18	0.2
25-29.9	4.6	3.74, 5.68	<0.001	4.32	3.45, 5.44	<0.001
≥ 30	13.7	11.1, 17.1	<0.001	16.5	13.0, 21.2	<0.001
History of Hepatitis B	1.21	0.60, 2.55	0.6	0.93	0.41, 2.20	0.9
History of Hepatitis C	0.64	0.39, 1.06	0.1	0.58	0.32, 1.05	0.1
Alcohol Use						
Never drink	*Ref*					
Drink daily	0.87	0.64, 1.18	0.4	1.5	1.03, 2.18	0.035
Drink few times/week	0.85	0.69, 1.06	0.2	1.24	0.95, 1.62	0.11
Drink few times/month	0.78	0.63, 0.96	0.021	0.97	0.74, 1.27	0.8
Drink few times/year	0.91	0.75, 1.11	0.4	1.05	0.82, 1.34	0.7
Moderate physical activity	0.5	0.43, 0.59	<0.001	0.69	0.57, 0.85	<0.001
Full food security	0.86	0.74, 0.99	0.039	0.75	0.61, 0.91	0.003
Private insurance	1.11	0.96, 1.27	0.2	1.17	0.97, 1.41	0.1
Access to healthcare	1.44	1.20, 1.72	<0.001	1	0.80, 1.26	>0.9

Married/Living with partner	1.42	1.23, 1.64	<0.001	1.28	1.07, 1.53	0.007

Supplementary Table 3A. Logistic regression model evaluating the association between education level, income bracket, and metabolic dysfunction-associated steatotic liver disease (MASLD), adjusted for sociodemographic factors (Model 1). *OR: Odds ratio; CI: Confidence interval.*

	**Univariable**			**Multivariable**		
	**OR**	**95% CI**	***p* value**	**OR**	**95% CI**	***p* value**
Education						
<College	*Ref*					
College or above	0.74	0.63, 0.86	<0.001	0.73	0.60, 0.87	<0.001
Income						
<65,000	*Ref*					
≥$65,000	0.97	0.84, 1.12	0.7	1.11	0.94, 1.30	0.2
Age	1.02	1.02, 1.03	<0.001	1.02	1.02, 1.03	<0.001
Male	1.4	1.22, 1.62	<0.001	1.35	1.17, 1.56	<0.001
Race/Ethnicity						
Non-Hispanic White	*Ref*					
Mexican American	1.74	1.38, 2.21	<0.001	1.95	1.53, 2.50	<0.001
Other Hispanic	1.12	0.85, 1.47	0.4	1.17	0.89, 1.54	0.3
Non-Hispanic Black	0.74	0.62, 0.89	0.001	0.78	0.64, 0.94	0.01
Non-Hispanic Asian	0.95	0.75, 1.21	0.7	1.15	0.89, 1.48	0.3
Other race	0.93	0.68, 1.27	0.6	1.06	0.77, 1.47	0.7

Supplementary Table 3B. Logistic regression model evaluating the association between income bracket, education level, and metabolic dysfunction-associated steatotic liver disease (MASLD), adjusted for covariates in Model 1 plus medical comorbidities (Model 2). *OR: Odds ratio; CI: Confidence interval; BMI: Body mass index.*

	Univariable			Multivariable		
	**OR**	**95% CI**	***p* value**	**OR**	**95% CI**	***p* value**
Education						
<College	Ref					
College or above	0.97	0.84, 1.12	0.7	1.1	0.92, 1.33	0.3
Income						
<65,000	Ref					
≥$65,000	0.74	0.63, 0.86	<0.001	0.72	0.58, 0.89	0.003
Age	1.02	1.02, 1.03	<0.001	1.02	1.01, 1.03	<0.001
Male	1.4	1.22, 1.62	<0.001	1.4	1.18, 1.67	<0.001
Race/Ethnicity						
Non-Hispanic White	Ref					
Mexican American	1.74	1.38, 2.21	<0.001	1.71	1.29, 2.26	<0.001
Other Hispanic	1.12	0.85, 1.47	0.4	1.1	0.80, 1.52	0.6
Non-Hispanic Black	0.74	0.62, 0.89	0.001	0.59	0.48, 0.74	<0.001
Non-Hispanic Asian	0.95	0.75, 1.21	0.7	2.4	1.77, 3.25	<0.001
Other race	0.93	0.68, 1.27	0.6	0.99	0.68, 1.45	>0.9
Hypertension History	2.4	2.06, 2.80	<0.001	1.33	1.09, 1.63	0.005
Diabetes History	3.31	2.62, 4.22	<0.001	1.73	1.31, 2.30	<0.001
Hypercholesterolemia History	1.81	1.56, 2.11	<0.001	1.08	0.89, 1.31	0.5
BMI Category						
18.5-24.9	Ref					
<18.5	0.28	0.07, 0.80	0.037	0.43	0.10, 1.24	0.2
25-29.9	4.6	3.74, 5.68	<0.001	4.32	3.45, 5.43	<0.001
≥ 30	13.7	11.1, 17.1	<0.001	17	13.4, 21.8	<0.001
History of Hepatitis B	1.21	0.60, 2.55	0.6	0.93	0.41, 2.20	0.9
History of Hepatitis C	0.64	0.39, 1.06	0.1	0.56	0.31, 1.02	0.1
Alcohol Use						
Never drink	Ref					
Drink daily	0.87	0.64, 1.18	0.4	1.51	1.04, 2.20	0.03
Drink few times/week	0.85	0.69, 1.06	0.2	1.22	0.94, 1.59	0.1
Drink few times/month	0.78	0.63, 0.96	0.021	0.96	0.73, 1.25	0.7
Drink few times/year	0.91	0.75, 1.11	0.4	1.05	0.82, 1.33	0.7
